# A conceptual framework to guide public oral health planning in Limpopo province

**DOI:** 10.4102/hsag.v24i0.1109

**Published:** 2019-09-23

**Authors:** Lawrence Thema, Shenuka Singh

**Affiliations:** 1 Discipline of Dentistry, School of Health Sciences, University of KwaZulu-Natal, Durban, South Africa

**Keywords:** Oral health, Policy, Priority, Budget, Incorporation, Integration

## Abstract

**Background:**

There is limited understanding of the complexities surrounding public oral health service delivery in South Africa and the resulting impact on oral health outcomes.

**Aim:**

This study aimed to identify the strengths and challenges in oral health decision-making within the public health sector and to propose a conceptual framework to guide oral health service delivery in the province.

**Setting:**

This study was performed in the Limpopo province.

**Methods:**

National and provincial health policy documents were reviewed to identify statements on oral health service delivery. A face-to-face, semi-structured interview was conducted with the Limpopo Provincial Manager of Department of Health, Oral Health Services. Data were collected on oral health policies and the organisational structure of public oral health services. A self-administered questionnaire was completed by five district managers of public oral health services to obtain data on the delivery of public oral health services in Limpopo province.

**Results:**

The results indicated that oral healthcare was not explicitly mentioned, included or referred to in the examined health policy documents. The interviews indicated that public oral health services do not have a dedicated budget and were not considered a priority. The questionnaire results revealed challenges in infrastructure, human resources and perceived marginalisation from the healthcare services. Participants agreed that there was a need for oral health to be clearly expressed and prioritised in health policy statements.

**Conclusion:**

This study proposed a framework that incorporated the identified core components that influenced oral health services provision in Limpopo province.

## Introduction

### Background

Despite the provision of free public oral health services in primary healthcare facilities within the Department of Health in Limpopo Province, South Africa (Department of Health 2015), common oral diseases, such as dental caries and periodontal disease, continue to impact the general health of individuals (Van Wyk & Van Wyk 2004). The oral health implications of communicable and non-communicable diseases are well documented, the resulting effect being an increased burden on public health services (Petersen 2008). The underlying determinants of ill health, such as malnutrition, unsafe drinking water, inadequate sanitation and exposure to infectious diseases, may further increase risk in contracting oro-facial-related diseases (Peterson 2008).

The Limpopo Provincial Health Department has made concerted efforts through policy and planning endeavours to improve oral health services, and there is commitment to ensure the availability of health services that are closely located to the communities served (Department of Health 2015); however, many challenges still exist. Oral health services remain largely located in urban areas with a focus on mainly curative services, such as dental fillings and extraction of teeth (Department of Health 2015).

While the Limpopo Province Oral Health Transformation Plan (2014–2019) recommended that prevention and management of common oral diseases in Limpopo province require an adequate number of oral health professionals in relation to the target population, there is little documented evidence of translation into practice (Singh, Myburgh & Lalloo 2010). The plan (2014–2019) also highlighted significant shortages and variation in the geographic distribution of oral health professionals and the availability of proper functioning dental facilities in the province. The lack of appropriate workforce and skills mix places further strain on under-resourced services (Singh et al. 2010).

This study therefore aimed to establish the extent to which public oral health services are expressed in selected national and provincial policy documents, and consequently implemented at provincial and district levels. The study also aimed to propose a conceptual framework to guide policy planning and development.

## Methods

This was a descriptive and exploratory study, conducted in all five districts of Limpopo province (Capricorn, Mopani, Sekhukhune, Vhembe and Waterberg). Data collection included oral health policy document review, semi-structured interviews with the provincial and district managers of public oral health services in Limpopo province, and a self-administered questionnaire for the latter.

Policy documents related to oral healthcare were retrieved from the National and Limpopo Department of Health’s websites. Policy documents examined included the *National Health Act* (Act 61 of 2003), Provincial Health Strategic Plan (2013/2014), Provincial Annual Performance Plan (2013/2014), Limpopo Province Oral Health Transformation Plan (2014–2019) and South African National Oral Health Strategy (2010). The districts were coded as follows: Capricorn – A, Mopani – B, Sekhukhune – C, Vhembe – D and Waterberg – E. Informed consent was obtained from all participants in writing before commencing the study, and issues of data security, confidentiality and privacy were maintained. Each document was reviewed to establish the nature and extent of any reference to oral health services.

A semi-structured interview was conducted with the Manager of Oral Health Services in the Provincial Department of Health, Oral Health Services to gain better understanding of provincial oral health policy development and implementation. The interview schedule focused on the following areas: existence of a provincial oral health policy or plan; monitoring of oral health policy implementation; strengths and limitations of the current oral health services; and recommendations for improving the provincial oral health services in Limpopo.

A self-administered questionnaire was used to collect data from the five district managers responsible for coordination of oral health service delivery in the identified districts. The questionnaire included questions on institutional support for oral health, organisational structure of oral health services and service delivery. The questionnaire made use of a Likert scale format that included scales ranging from strongly disagree = 1 to strongly agree = 5.

A follow-up, semi-structured interview was held with each district manager after the questionnaire was completed to gain better understanding of their perspectives on oral health service delivery. The interview schedule focused on priority of oral health programmes in the district; existence of policy guidelines for oral health service delivery at district level; monitoring and evaluation of policy guidelines; availability of resources for oral health service delivery; strengths and challenges of oral health service delivery; and recommendations for improved oral health services. All interviews were digitally recorded once permission was obtained from each participant.

### Data collection and analysis

The policy documents were analysed using content analysis.

Data from the questionnaire were analysed using SPSS version 23.0 (IBM Corp., Foster City, CA). The responses to the open-ended questions were grouped, and emergent themes were examined and compared for possible associations.

The data from the interviews were transcribed verbatim, and the narratives for each interview were organised and examined for emergent themes (Braun & Clarke 2006). Each compiled narrative was sent to the respective respondent to verify the accuracy of the recorded information, and participants also had an opportunity to comment on the analysed data.

### Validity and reliability

A pilot study was conducted with 10% of participants (not used in the study) to review and refine the questions posed in the questionnaire. The research proposal and the research instrument was also reviewed by a peer-review panel located within the institution where the researchers are based. Content validity was maintained by ensuring that the domains of the questionnaire were relevant to the study population’s work responsibilities and interests.

Reliability of the data was determined by ensuring that the data was double-checked during data entry and all outliers were corrected. Internal validity was maintained by ensuring a logical structure in the research report with clearly defined methods to execute the study. External validity focused on the generalizability of the study findings.

### Ethical considerations

Ethical clearance was obtained from the Biomedical Research Ethics Committee of the University of KwaZulu-Natal (BREC REF: 327/14), and approval to conduct the study was obtained from the Department of Health in Limpopo Province.

## Results

The extent to which oral healthcare is expressed in national and provincial policy documents is presented in Table 1. The *National Health Act* made no reference to oral health.

**TABLE 1:** National and provincial policy documents reviewed.

National and provincial health policy documents	Findings
*National Health Act* 61 of 2003	No specific reference to oral health.
South African National Oral Health Strategy 2010	This is the main policy document driving oral healthcare in South Africa. Despite its strong content, there was no expressed guidance on the translation of oral health policy into implementable programmes at provincial and district levels.
Provincial Health Strategic Plan (2013–2014)	No indication of an oral healthcare plan, or the inclusion of oral healthcare in general health promotion initiatives.
Provincial Annual Performance Plan (2013–2014)	No specific reference to oral health.
Limpopo Province Oral Health Transformation Plan (2014–2019)	There is reference to an oral healthcare plan for the province.

The results of the interview with the Provincial Manager of Oral Health Services revealed challenges in the implementation and monitoring of oral health policy in Limpopo province (Table 2). The provincial manager indicated a disconnect between national and provincial oral health planning, where ‘national promises to assist us to amend our provincial one; we are creating our own and the province has its own standardization; so it becomes a challenge’ (Provincial manager).

**TABLE 2:** Responses from interview with the provincial manager.

Interview questions	Provincial manager’s responses
**A. Policy** Q.1: Is there an overall oral health policy? Themes arising: Lack of policy commitment Lack of dedicated budget	‘We have a provincial draft, we are working on a draft; just like national is working on a draft.’ (Provincial manager) ‘There is very little oral health, just like in the Annual Performance Plan, up until now there is no oral health even in the strategic planning. There is not even a budget for oral health. Allied services, the physiotherapists and dieticians are included, but there is no clear picture of where oral health is.’ (Provincial manager)
**B. Monitoring** Q 2: How is monitoring of policy implemented? Themes arising: Limited oral health indicators Limited focus on oral healthcare	‘There is also monitoring and evaluation in the hospitals. Oral health again is not there.’ (Provincial manager) ‘The only indicators that we had, are at primary, oral health indicators, the fissure sealants, extractions.’ (Provincial manager) ‘We are creating our own and the province has its own standardization; so it becomes a challenge. Evaluation and health indicators, that helps to improve the quality of services.’ (Provincial manager)
What are the strengths and limitations of the services? Themes arising: Lack of support for oral healthcare Oral health is a non-priority	‘I think oral health is battling with a lot of attitude; there are very few hospitals where oral health gets support and is regarded as part of the hospital. Oral health is represented by the medical clinical manager, and when they have executive meetings, oral health is largely forgotten.’ (Provincial manager)

The results from the questionnaire indicated that district managers were having challenges for oral health service delivery due to a lack of ‘provincial oral health policy’ (Table 3). Other challenges included ‘no dedicated oral health budget, challenging procurement for basic consumables and repair and service of equipment’ (Participants A, B, C, D and E). In the follow-up interview, district managers identified the following challenges: oral health being low on the policy agenda, lack of dedicated budget for oral health services and poor infrastructure for oral health services (including mobile oral health services) (Participants A, B, C, D and E).

**TABLE 3:** Responses from district managers.

District managers’ questions	Responses to questionnaire and follow-up interviews
A. Institutional support for oral health Are there policy guidelines for oral health?	Five managers indicated that there was no known policy for providing services or implementing district oral health policy guidelines. Each institution followed its own guidelines.
How are the policy guidelines monitored? Themes arising: Utilisation reports Appropriate services	Three managers were unaware of any policy monitoring procedures in their districts.
Are oral health programmes given priority by the health authorities? Themes arising: Dedicated budget	Four managers disagreed that oral health programmes were given priority by the health authorities, as there was no dedicated budget for oral health.
Budget allocations for infrastructure and equipment? Themes arising: Infrastructure Equipment Resources Oral health professionals.	Four managers strongly disagreed that there were budget allocations for infrastructure and equipment. Five managers indicated a lack of resources. Two managers agreed that there was adequate infrastructure for oral health services. Five managers agreed that there was lack of sufficient dental equipment and appropriate oral health personnel.
There are enough resources to perform and manage our duties with ease.	Only four managers agreed that there were adequate resources to perform and manage duties with ease.
What are the challenges for oral health service delivery? Themes arising: Inadequate management posts Procurement Repair and service equipment Enough rooms Support from centre manager Transport	The respondents noted the following: ‘Not having the provincial oral health policy.’ (Participant A, B, C, D and participant E) ‘Lack of recognition of the oral health profession and its purpose.’ (Participant A, B, C, D and participant E) ‘The failure to create management posts at strategic levels.’ (Participant A, B, C, D and participant E) ‘No dedicated budget for oral health.’ (Participant A, B, C, D and participant E) ‘Challenging procurement for basic consumables and repair and service of equipment.’ (Participant A, B, C, D and participant E) ‘Non-functional dental equipment, not enough rooms.’ (Participant A, B, C, D and participant E) ‘Lack of support from centre manager for logistical and transport.’ (Participant A, B, C, D and participant E)

## Discussion

This study examined the possible impact of policy, and its implementation (and lack thereof) at various levels of oral service provision in Limpopo province. The study also identified the specific levels (national, provincial and district) at which strengths and challenges of oral healthcare were indicated.

### Policy

Overall document analysis indicated that oral health expressions or statements are not uniformly included in the identified health policy documents. The results suggested a non-prioritisation of oral health, lack of implemented oral health policy and lack of dedicated budget for oral health. The provincial manager indicated that the provincial oral health policy was still in draft form. This reported lack of formalised oral health policy or uniform policy guidelines implied that communities in Limpopo province could be disadvantaged in accessing equitable oral health services. This finding is consistent with Singh et al. (2010), who also reported that oral health policy, planning and implementation were not consistent in all provinces in South Africa. The results further suggested that oral health service delivery in Limpopo province was not integrated with other key health services. This disadvantages oral health services not only by its exclusion from policy documentation, but through alienation from health teams’ joint activities (Thema & Singh 2013).

### Strategic planning

The findings of the present study support those of Singh et al. (2010), which revealed limited communication, collaboration or partnership in planning for primary healthcare and implementing healthcare practice (Singh et al. 2010). Effective partnership development and collaborative efforts in oral health should avoid duplication of services and promote increased access to care (AACDP 2010; Santa Fe Group 2008; Sheiham 2005). The study findings also support other studies that front-line primary healthcare professionals (namely, community nurses, community medical doctors and their assistants) should prioritise vulnerable and underserved communities (Sheiham 2005; Thema & Singh 2013). Primary healthcare professionals could be instrumental in providing screening services for oral diseases, and strategies could include the incorporation of oral health education into the day-to-day service provision. This, however, can only be possible, if there is regular and ongoing in-service training for health professionals to better equip them with skills in identifying and referring patients or clients with oral conditions (OHCC 2014; Sheiham 2005). The envisaged inter-professional collaborative practice would increase the number of healthcare settings that provide oral healthcare in health centres (Thema & Singh 2013). Oral health professionals have the opportunity to detect chronic conditions that share risk factors with oral diseases, specifically hypertension and diabetes, while providing oral healthcare (AACDP 2006; OHCC 2014).

### Budget

The results indicated that the lack of dedicated budget negatively impacts oral healthcare. This finding is consistent with other studies examining oral health service in Limpopo province (Thema & Singh 2017). Limited resources have a wide-ranging negative impact on health services in both developing and developed countries (AACDP 2010; Mejia et al. 2008; USDoHHS 2014). Similarly, lack of dedicated budget was also observed in Hispanic communities, which resulted in limited infrastructure, fewer professionals, limited consumables, and limited transport and maintenance for mobile oral health services (Mejia et al. 2008). By South African standards, lack of dedicated budget ultimately results in violation of the Constitution of the Republic of South Africa, Batho Pele Principles and the South African Patient’s Charter of 1999, which collectively advocate individual rights to healthcare.

Noting the current challenges with oral planning in the province, the following framework is proposed to guide policy monitoring and review. This framework comprises the following components: prioritisation of oral health, the role of policy, budgetary allocations, need for strategic planning and organisation within health services, and collaboration and support from management. These identified components are similar to other frameworks developed for vulnerable populations in developing countries and underserved communities (AACDP 2006; Bhayat & Cleaton-Jones 2003; Magnussen, Ehiri & Jolly 2004; OHCC 2014; Petersen 2008).

### The proposed framework for oral health planning

**FIGURE 1:**
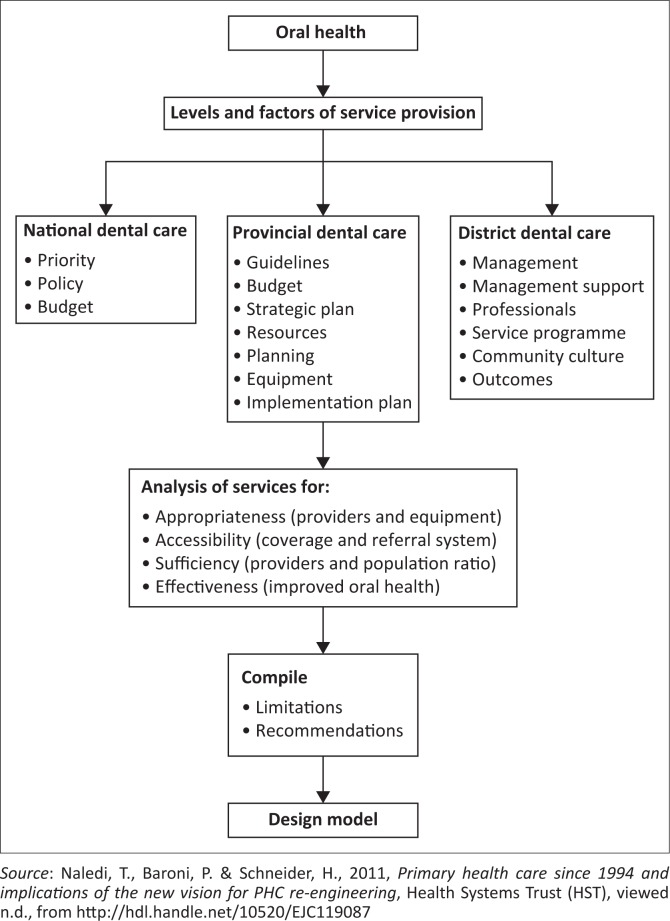
Framework for oral health planning.

The framework described below focuses on the levels of provision of oral healthcare, aspects that determine and influence the status of services, and their relationship in impacting public oral health services provision in Limpopo province (Ngulube, Mathipa & Gumbo 2015). The determinants of public oral health services provision in Limpopo province may be categorised into groups emanating from different levels of government services provision. Their responsibilities, which include appropriate, accessible and effective services, are highlighted within Figure 1 (Naledi, Baroni & Schneider 2011). The determinants may be classified as macro-factors, emanating at national government level, where services of priority are determined, policy and services guidelines are formulated, and considered in the health budget (Naledi et al. 2011).

The next group of determinants are located at the provincial level, and they focus on policy adoption or translation and budgetary allocations, in addition to the identification of resources to build capacity in programme delivery.

Overall, Naledi et al. (2011) advocate development of an oral health policy and unequivocal service guidelines to be incorporated in the health policy document. This incorporation would enhance prioritisation of oral health and consideration in the health budget. Consideration and inclusion of oral health in the health budget could have a positive impact on oral health resources, strategic planning, implementation and evaluation of services. The purpose of this framework is to highlight the need for appropriate skills mix in oral health human resources to deliver optimal oral healthcare, improve access for oral health and ensure sustainability of service provision.

On a practical level, the components highlighted in the conceptual framework could assist oral health planners in identifying the key strategic areas that could determine the successful implementation of national oral health policy. Human resource allocation, such as staffing and skills mix, together with budgetary infrastructure for facility and community-based oral healthcare needs to be clearly identified and supported at all levels of provincial health planning. It is equally important that oral health service delivery is monitored and evaluated using the identified key elements such as appropriateness of services provided, accessibility of oral healthcare and effectiveness of oral health services rendered. This requires close monitoring of the actual services provided so that challenges in service delivery can be easily identified and rectified.

### Strengths and limitations of the study

This study provided a clear understanding of the challenges facing oral health planners in the province. The proposed framework could be used to identify key areas for oral health policy development and implementation. One limitation of this study is that the framework still needs to be tested to determine its practicality. More research is needed in this area. Another limitation could be the generalisability of the study findings. Despite this limitation, the framework could be a useful tool in health planning in general.

## Conclusion

The results of the study indicate that oral health planning in Limpopo province needs more support from a policy and budgetary perspective. Resolving the challenges associated with policy development and budget allocations could contribute to improved oral health service delivery.
